# MicroRNA-154-5p regulates the HPV16 E7-pRb pathway in Cervical Carcinogenesis by targeting CUL2

**DOI:** 10.7150/jca.45871

**Published:** 2020-07-11

**Authors:** Weihong Zhao, Yatao Liu, Lili Zhang, Ling Ding, Yaqin Li, Honglei Zhang, Tong Wang, Min Hao

**Affiliations:** 1 Department of Obstetrics and Gynecology, the Second Hospital of Shanxi Medical University, Taiyuan 030001, China.; 2 Department of Health Statistics, School of Public Health, Shanxi Medical University, Taiyuan, Shanxi, 030001, China.

**Keywords:** cervical cancer, CUL2, invasion, miR-154-5p, pRb, proliferation

## Abstract

Cervical cancer, induced by persistent HPV infection, has a high mortality rate. The E3 ubiquitin ligase Cullin 2 (CUL2) is critical for HPV16 E7-mediated degradation of retinoblastoma protein (pRb). Dysregulation of microRNAs (miRNAs) is induced during tumorigenesis; however, the association between miRNA networks and CUL2, specific to cervical cancer, remains unknown. Herein, we determined miRNA profiles in cervical cancer tissues using an Affymetrix miRNA array. We found that miR-154-5p was downregulated during cancer progression using real-time quantitative reverse transcription PCR in 130 biopsy specimens. Bioinformatics analysis and dual-luciferase reporter assays indicated that miR-154-5p directly targets the CUL2 3'UTR. To determine the functional consequences of modulating miR-154-5p and CUL2 levels, HPV16-positive cervical cancer cell line (SiHa) was transfected with miR-154-5p mimic, miR-154-5p inhibitor, or CUL2 siRNA. The proliferation, migration, and invasion of transfected cells were evaluated using CCK8 cell counting kit, wound-healing assay, and Transwell invasion assay. Increased miR-154-5p expression promoted significantly reduced SiHa cell proliferation, migration, and invasion, whereas the miR-154-5p inhibitor had the opposite effect. CUL2 silencing had similar effects to those of the miR-154-5p mimic. Consistent with the inverse correlation between miR-154-5p and CUL2 levels, CUL2 silencing also increased pRb expression. To our knowledge, this is the first study to demonstrate that miR-154-5p regulates pRb expression by targeting CUL2 3'UTR, thereby playing a tumor-suppressive role in HPV16 E7-induced cervical carcinogenesis.

## Introduction

In China, cervical cancer has the highest incidence and mortality levels among female reproductive tract malignancies 1. Squamous cell carcinoma (SCC), which is the predominant histological type, accounts for 80-85% of all cervical cancer cases. SCC develops from pre-existing non-invasive squamous precursor lesions referred to as cervical intraepithelial neoplasia (CIN). It has been confirmed that persistent infection with high-risk human papillomavirus (HR-HPV) is the primary cause of cervical cancer, and HPV16, in particular, is associated with more than 50% of SCC cases 2. Although CIN can either progress or regress 3, only 1% of the HPV-infected individuals might develop cervical cancer 4. Therefore, the molecular mechanisms involved in HPV16 infection to the development of CIN and eventually to SCC remain largely unknown.

It is well established that HPV16 E7-dependent degradation of the retinoblastoma protein (pRb) is a central carcinogenic pathway. Owing to a lack of intrinsic enzymatic activity, HPV16 E7 relies on protein-protein interactions to modulate the pRb degradation, especially interactions with the Cullin 2 (CUL2) ubiquitin ligase 5, 6. Huh *et al.* found that HPV16 E7 binds to CUL2 and promotes pRb degradation, thereby causing malignant transformation of cells 5, while Todorovic *et al.* confirmed that the conserved region 3 of HPV16 E7 is a critical key for pRb degradation and CUL2 activity, supporting the finding that E7 and CUL2 interaction results in pRb degradation and leads to cell cycle alterations 7. Therefore, CUL2-mediated regulation of HPV16 E7-induced pRb degradation is a critical step for the progression of HPV16 infection into cervical cancer.

miRNAs downregulate approximately a third of protein-coding genes *in vivo* through translational repression or gene silencing and are involved in various stages of viral infection and tumorigenesis 8. However, reports on the correlation between miRNA expression and CUL2 regulation in the context of cervical cancer are rare. Our present study identified that miR-154-5p was significantly downregulated in SCC and its levels were negatively correlated with the proliferation and metastasis of SiHa cells. Mechanistic analyses revealed that miR-154-5p plays a tumor-suppressive role in cervical cancer through mediating pRb expression by targeting CUL2 3'UTR. Therefore, miR-154-5p may be a potential therapeutic target to block the progression of CIN into SCC caused by persistent HPV16 infections.

## Materials and Methods

### Clinical specimens

**Figure 1:**
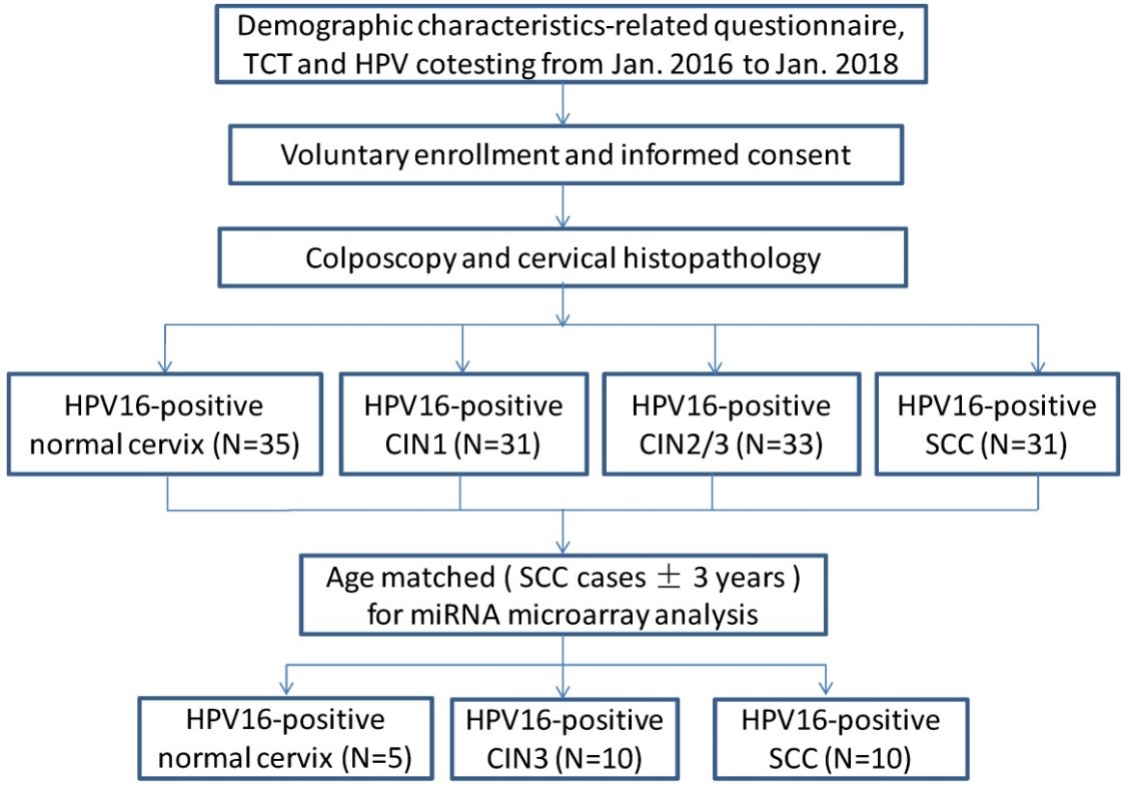
Study flow diagram.

A total of 130 human cervical biopsy specimens including those from patients with only HPV16-positive normal cervix (n=35), only HPV16-positive CIN1 (n=31), only HPV16-positive CIN2/3 (n=33), and only HPV16-positive SCC (n=31) were collected at the Second Hospital of Shanxi Medical University between January 2016 and January 2018. According to the 2009 International Federation of Gynecology and Obstetrics (FIGO) staging standard, there were 12 cases of stage Ia, 7 cases of stage Ib, and 12 cases of stage IIa cancers. According to pathological classification, there were 9 cases of well-differentiated, 20 cases of moderately differentiated, and 2 cases of poorly differentiated cancers. 25 age-matched samples (SCC cases±3years) including 10 SCC tissues, 10 CIN3 tissues and 5 normal cervix tissues were selected out of total 130 patients for miRNA microarray analysis (Figure 1, see Table 1). All participants were Han Chinese who have lived in Shanxi for more than 5 years. Tumorigenesis was confirmed by two pathological examinations. Participants with leukemia, liver disease or other tumors were excluded. No patient received radiotherapy or chemotherapy before the tissues were obtained. After obtaining written informed consent from all participants, uterine cervix tissue biopsies were obtained by colposcopy, and the samples immediately stored in a -80 °C freezer. This study was approved by the Ethical Committees of the Second Hospital of Shanxi Medical University (IRB number: 2013-002).

### MicroRNA microarray analysis and bioinformatics analysis

Total RNA was extracted using Trizol® reagent (Life Technologies, Carlsbad, CA) and purified with an RNeasy® mini kit (Qiagen, Valencia, CA). Biotinylated cDNA was prepared from 250 ng of total RNA using the Ambion® WT Expression Kit (Affymetrix, Santa Clara, CA) according to the standard Affymetrix protocol. Following biotinylation, fragmented cDNA was hybridized for 16 h at 45 °C onto a GeneChip® miRNA 4.0 Array (Affymetrix, Santa Clara, CA). GeneChips were washed and stained in the Affymetrix Fluidics Station 450. All arrays were scanned using the Affymetrix® GeneChip Command Console, which was installed in the GeneChip® Scanner 3000 7G.

The prediction of miRNA target genes was performed using two public algorithms and associated databases (miRbase, available at http://mirbase.org; and TargetScan version 7.1, available at http://targetscan.org). Gene ontology (GO) analysis was applied to analyze the main function of the target genes that had been classified according to the GO Project. In addition, pathway analysis was used to identify the key pathways of target genes according to the Kyoto Encyclopedia of Genes and Genomes (KEGG). Notably, candidate miRNAs that could potentially bind to the CUL2 3'UTR were identified, and binding affinity scores were calculated using TargetScan version 7.1.

### Dual-luciferase report assay

The CUL2 3'UTR wild type fragment (CUL2-WT) containing the predicted binding site of miR-154-5p and its corresponding mutant (CUL2-MUT), and the miR-154-5p inhibitor sponge (PC) were cloned by PCR into the pmirGLO vector (Promega, Madison, WI). The putative miR-154-5p binding site was mutated in the CUL2-MUT sequence by replacing 5'-AATATGACAGATAACCT-3' with 5'-TTTAACACACTATTGGA-3'. 293T cells were co-transfected with pmirGLO-CUL2-WT, pmirGLO-CUL2-MUT, or pmirGLO-PC plus either miR-154-5p mimic negative control (NC). After incubating for 24 h and 48 h, cells were collected for Firefly and Renilla luciferase activity measurements using the dual-luciferase reporter system (Promega, Madison, WI).

### Cell lines and transfection

Two HPV16-positive human cervical cancer cell lines (SiHa and Caski) and 293T cells were purchased from the Cell Center of Shanghai Institutes for Biological Sciences. All cell lines were authenticated by the above-mentioned cell center by means of short-tandem repeat-polymerase chain reaction profiling. The cells were maintained in DMEM (Gibco, Carlsbad, CA) supplemented with 10% FBS (Gibco). SiHa cells were transfected with miR-154-5p mimic, miR-154-5p mimic NC, miR-154-5p inhibitor, miR-154-5p inhibitor NC, CUL2 siRNA and CUL2 siRNA NC using Lipofectamine 3000 (Invitrogen, Carlsbad, CA) in accordance with the manufacturer's protocol. Blank control (where only the transfection reagent was added) and mock control were set up in all experiments. All nucleotides were FAM-labeled from GenePharma (Shanghai, China; see Table 2).

### Real-time quantitative reverse transcription PCR (RT-qPCR)

Total RNA was isolated from cervical tissues or cell lines using the Eastep® Super Total RNA Extraction Kit (Promega, Madison, WI). cDNA was synthesized using Oligo (dT) and specific stem-loop primers with the PrimeScript^TM^ RT reagent Kit (Takara, Kusatsu, Japan) added to a SYBR Premix containing Ex Taq^TM^ (Takara, Kusatsu, Japan). The relative expression of miR-154-5p was calculated using the 2^-ΔΔCt^ method with H-U6 as endogenous control. All specific primers were purchased from Sangon Biotech (Shanghai, China; see Table 2).

### Cell proliferation assay

SiHa cells were seeded into a 96-well plate. At the indicated time post-seeding (0, 48, 96 h), 10 μl of Cell Counting Kit-8 (CCK-8; Dojindo Laboratories, Kumamoto, Japan) solution was added to each well. After further incubation for 1.5 h, absorbance at 450 nm was recorded using a spectrophotometer (BioTek, Winooski, VT).

### Wound-healing assay

SiHa cells were seeded into 6-well plates until they reached 90% confluency. A straight scratch was uniformly made in the center of each well with a micropipette tip. After scratching, cells were gently washed with PBS to remove the detached cells and maintained in serum-free DMEM. Cell migration was photographed and evaluated at 24 h and 48 h after scratching by light microscopy (Olympus, Tokyo, Japan).

### Transwell invasion assay

Matrigel was diluted 1:5 with serum-free DMEM and added to the Transwell chambers (BD, Franklin Lakes, NJ) for incubation 3 h. The cells were starved in serum-free DMEM for 12 h before 2×10^4^ cells were seeded to each upper chamber inserted in a 24-well plate. 500 μl of DMEM with 10% FBS was added to the lower chambers. After incubating for 24 h, the noninvasive cells on the upper membrane were wiped with a cotton swab, and the invaded cells on the underside were fixed in methanol and stained with 0.1% crystal violet, then counted under a light microscope.

### Western blot analysis

At 72 h following transfection, SiHa cells were collected and total protein extracted using RIPA lysis buffer (Beyotime Biotechnology, Shanghai, China). Conventional western blot analysis was performed using the primary antibodies anti-CUL2 (1:200, mouse; santa cruz; sc-166506), anti-pRb (1:200, mouse; santa cruz; sc-102) or anti-β-actin (1:1,000, mouse; santa cruz; sc47778). After adding HRP-linked donkey anti-mouse IgG (1:2,000; abcam; ab97030), binding was detected using the Western Lightning Plus ECL reagent (Waltham, MA).

### Statistical analysis

For microarray analyses, we used Student's *t*-test in the Limma R package (version 3.36.5) to filter for differentially expressed miRNAs. Empirical Bayesian moderation was used to correct the *P* values. The threshold set was a fold change of > 2.0 and *P*<0.05. The Fisher's exact test and Chi-square test were used to select the significant GO and pathway. The threshold set was *P*<0.05. Each *in vitro* experiment was performed in triplicate, and the results were presented as the mean value ± standard error of mean. All statistical analyses were performed using the SPSS 16.0 software (IBM, Chicago, IL). Analysis of variance (ANOVA) or Kraskal-Wallis test with Post-hoc comparisons were used to establish the differences; *P*<0.05 was considered statistically significant.

## Results

### miRNA expression profiles in different cervical tissues and bioinformatics analysis

**Figure 2:**
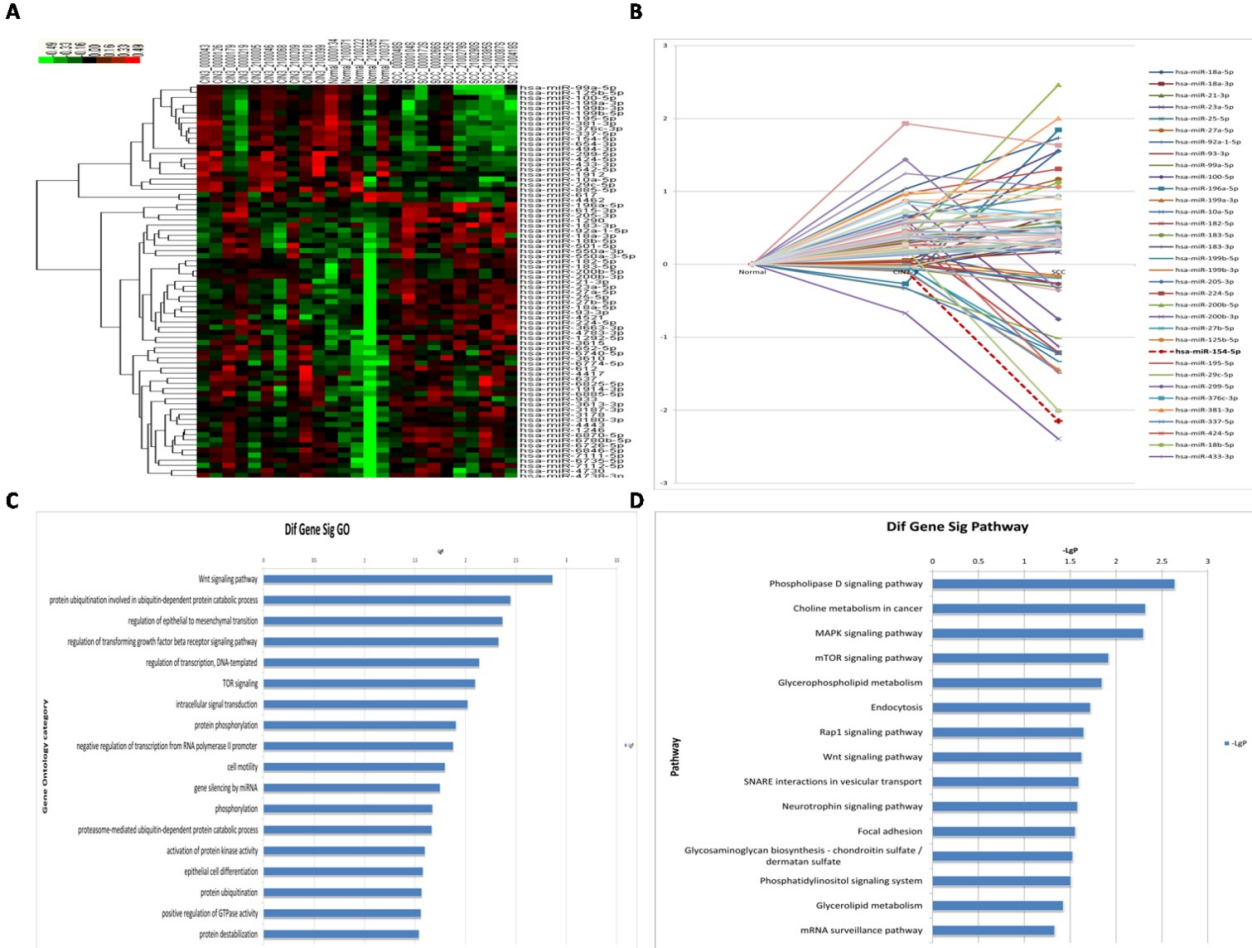
** miRNA profiles in different HPV16 positive cervical tissues.** (A) Hierarchical cluster of miRNA profiles. (B) The dynamic expression characteristics of miRNA profiles. (C) Log histogram of the significant GO. (D) Log histogram of the significant Pathway.

A total of 77 miRNAs were differentially expressed in the three groups (Figure 2A, 2B, see Supplementary Table 1). The 77 differentially expressed miRNAs were searched in both miRbase and TargetScan to predict their target genes, and overlapping genes were used as the final dataset. A total of 2,618 target genes were predicted. GO analysis for highly enriched gene pathways identified the Wnt signaling pathway, the protein ubiquitination pathway (specifically, ubiquitin-dependent protein catabolic processes), the transforming growth factor beta receptor signaling pathway, as well as genes involved in regulation of epithelial to mesenchymal transition and regulation of transcription (Figure 2C). Moreover, KEGG analysis of the highly enriched gene pathways identified the phospholipase D signaling pathway, MAPK signaling pathway, mTOR signaling pathway, as well as genes involved in choline metabolism in cancer, and in glycerophospholipid metabolism (Figure 2D).

### CUL2 is a direct target of miR-154-5p

**Figure 3:**
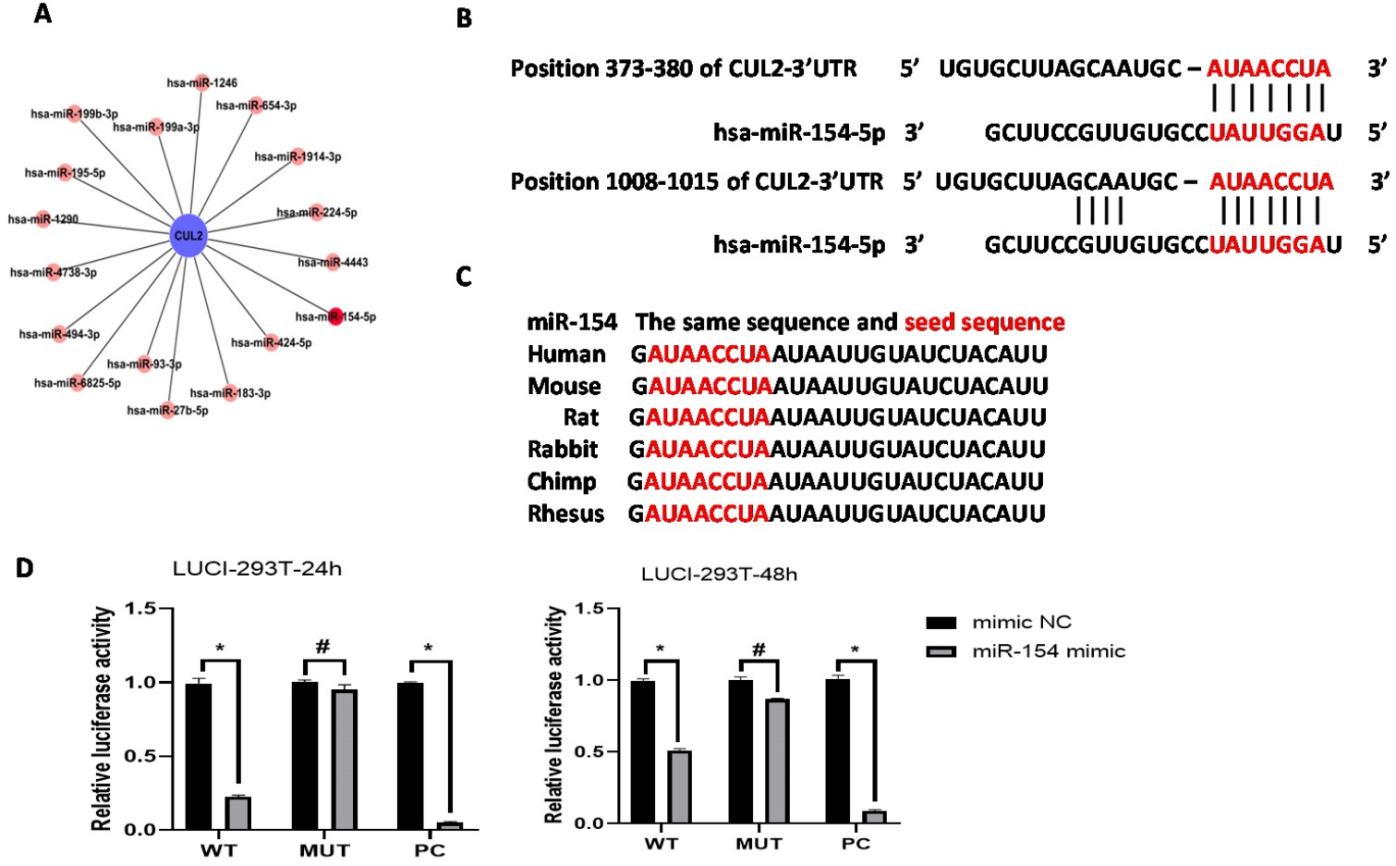
** miR-154-5p can bind to CUL2 3'UTR directly.** (A) Bioinformatics analysis predicted that CUL2 3'UTR was potentially targeted by 17 differentially expressed miRNAs. (B) The putative binding sites of CUL2 3'UTR and miR-154-5p. (C) miR-154-5p sequence is highly conserved. (D) Dual-luciferase report assays of cells cotransfected with miR-154-5p mimic and wild-type or mutant luciferase reporter. (*, P<0.05 versus corresponding NC).

Bioinformatics analysis predicted that the CUL2 3'UTR could potentially be targeted by the 17 differentially expressed miRNAs; among them, the miR-154-5p had the highest context percentile score (Figure 3A, see Table 3). miR-154-5p can potentially bind to two regions on CUL2 (Figure 3B), and it is highly conserved among different species (Figure 3C). These results suggest that miR-154-5p can form stable and close bonds with the CUL2 3'UTR.

Further, the dual-luciferase reporter assay showed that cells co-transfected with the miR-154-5p mimic and CUL2-WT plasmids had significantly less relative luciferase activity than cells co-transfected with the mimic NC and CUL2-WT plasmids (*P*<0.05), whereas cells co-transfected with the miR-154-5p mimic and CUL2-MUT plasmids showed no significant reduction in the luciferase signal compared to the mimic NC (*P*>0.05). Together, these results confirmed the direct binding between miR-154-5p and CUL2 (Figure 3D).

### Expression of miR-154-5p in different cervical tissues and cells

**Figure 4:**
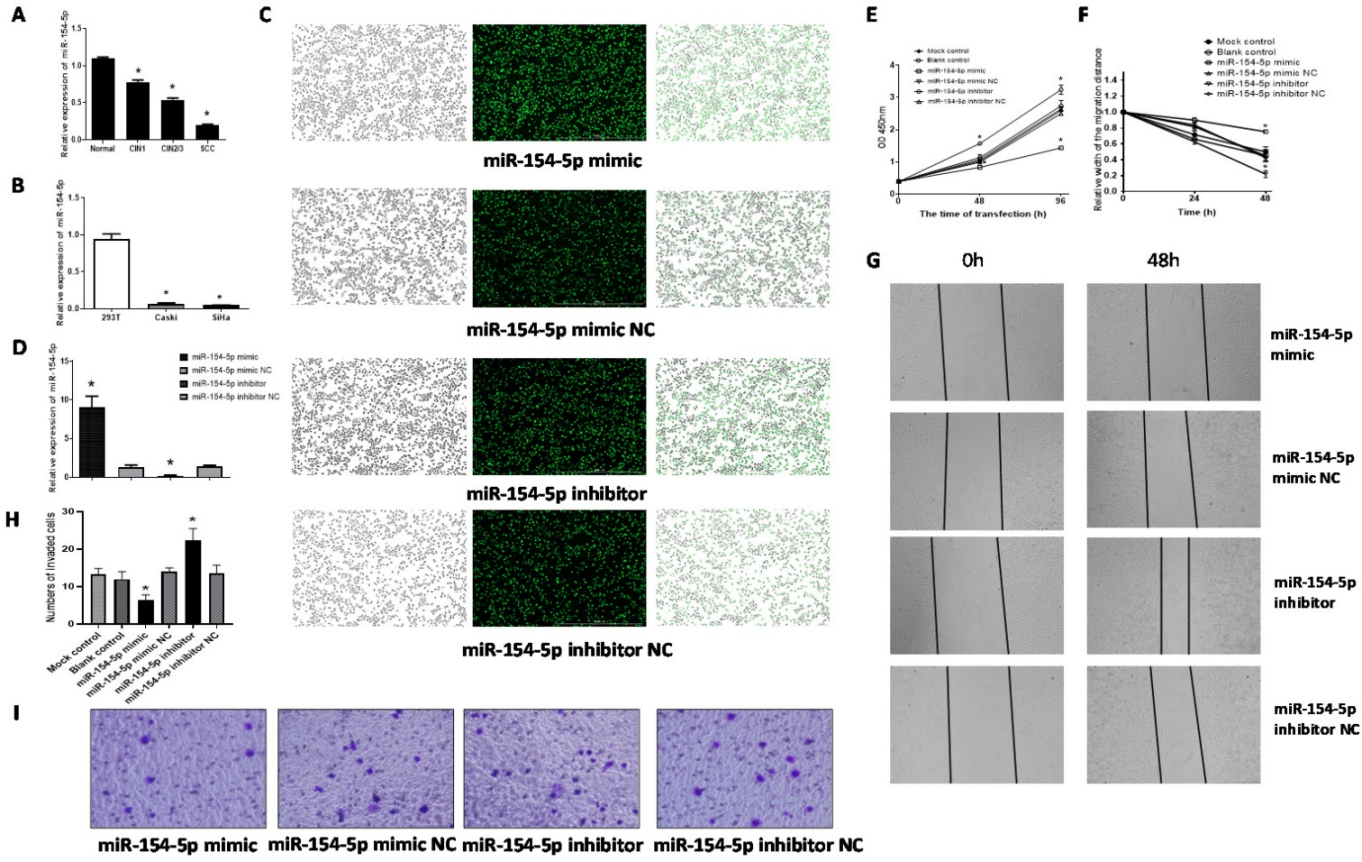
** miR-154-5p was downregulated in cervical cancer tissues and effected SiHa cells proliferation, migration, and invasion.** (A) miR-154-5p was downregulated in CIN and SCC tissues. (B) miR-154-5p was downregulated in cervical cancer cells. (C) SiHa cells were transfected with miR-154-5p mimic/inhibitor and corresponding NC. (D) Relative expression of miR-154-5p measured using RT-qPCR 48h post transfection. (E) miR-154-5p effected the SiHa cells proliferation. (F-G) miR-154-5p effected the SiHa cells migration. (H-I) miR-154-5p effected the SiHa cells invasion. (*, P<0.05 versus corresponding NC).

The level of miR-154-5p expression in the CIN1, CIN2/3 and SCC were all lower than that in the normal cervix (F=129.83, *P*<0.001), consistent with the miRNA microarray analyses results (Figure 4A). Further, miR-154-5p levels were also lower in the two human cervical cancer cell lines (SiHa and Caski) compared with 293T (Figure 4B).

### miR-154-5p suppressed proliferation, migration, and invasion of SiHa cells

We used the miR-154-5p mimic or miR-154-5p inhibitor to transfect SiHa cells, and assessed the effect on proliferation, migration, and invasion. Transfection efficiencies were verified using fluorescence microscopy and by using RT-qPCR for the miR-154-5p levels. Fluorescence microscopic detection indicated transfection efficiencies over 90% in all groups (Figure 4C). The RT-qPCR results showed that miR-154-5p level was significantly increased following transfection with miR-154-5p mimic compared with mimic NC, while significantly decreased following transfection with miR-154-5p inhibitor compared with inhibitor NC (both *P<*0.001) (Figure 4D).

Augmentation of miR-154-5p after transfection with its mimic reduced SiHa cells proliferation whereas miR-154-5p silencing increased cell proliferation, compared with the corresponding NC groups (both *P<*0.05, Figure 4E). Transfection with the miR-154-5p mimic inhibited the SiHa cells migration, whereas transfection with the miR-154-5p inhibitor enhanced cell migration compared to transfection with the corresponding NCs (both *P<*0.05, Figure 4F, 4G). Compared with the corresponding NC, the invasive ability of SiHa cells transfected with miR-154-5p mimic decreased by 54.76%, while the invasive ability of SiHa cells transfected with miR-154-5p inhibitor increased by 63.41% (both *P* < 0.05, Figure 4H, 4I).

### miR-154-5p mediated pRb expression by targeting CUL2

**Figure 5:**
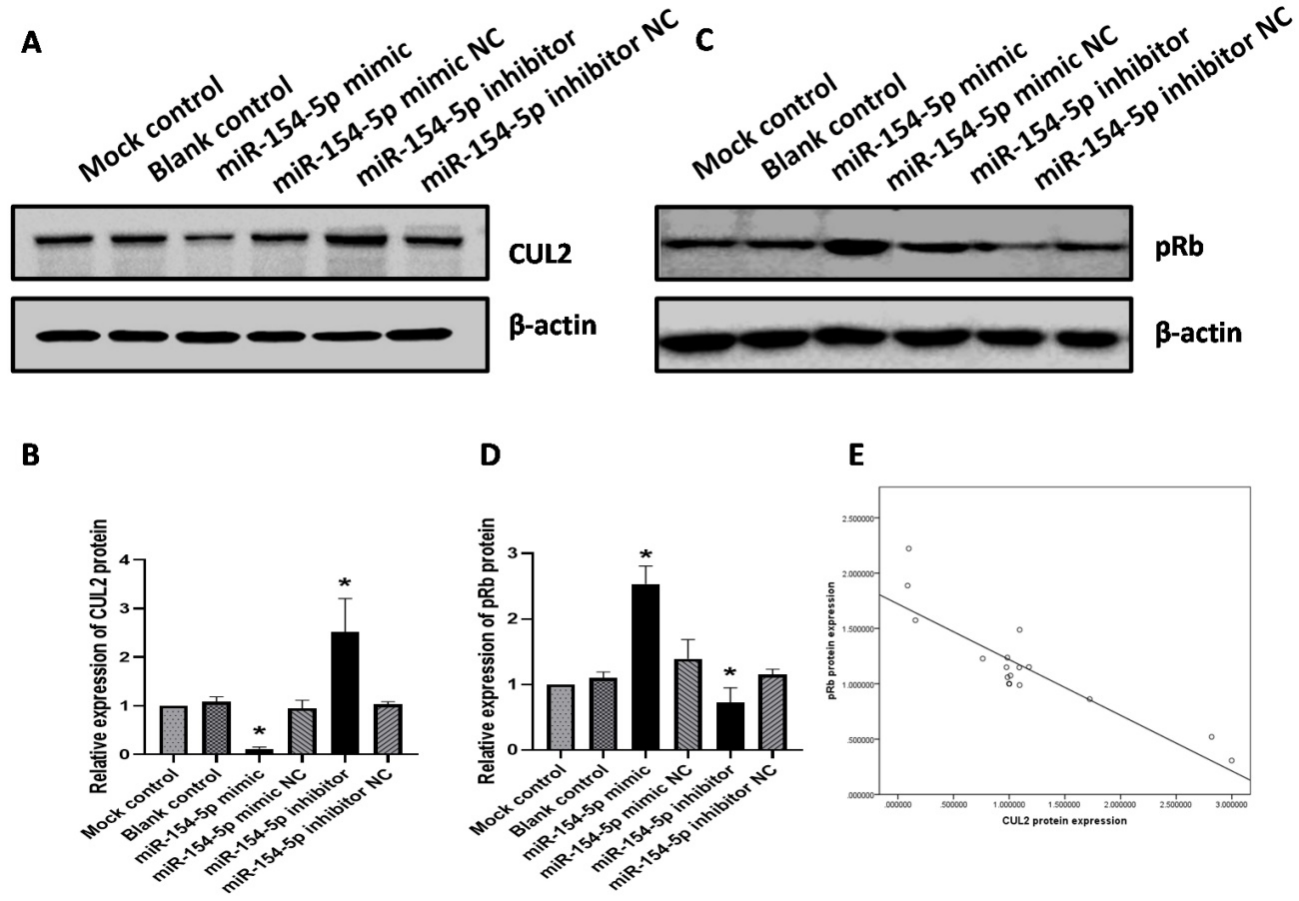
** miR-154-5p effected the expression of CUL2 and pRb.** (A-B) miR-154-5p regulated the expression of CUL2; (C-D) miR-154-5p regulated the expression of pRb; (E) Correlation analysis between CUL2 and pRb expression levels in SiHa. The relative expression of protein was measured by gray value assays. (*, P<0.05 versus corresponding NC).

As described earlier, miR-154-5p can bind directly to the 3'UTR of CUL2. To further elucidate whether miR-154-5p would exert an inhibitory effect on CUL2 expression, western blot analysis was performed on transfected SiHa cells. At 72 h post-transfection, the expression of CUL2 protein in cells transfected with miR-154-5p mimic was lower than that of the mimic NC, while the expression of CUL2 protein in the miR-154-5p inhibitor-treated group was higher than inhibitor NC (both *P<*0.05, Figure 5A, 5B). In direct contrast to the changes observed with CUL2 expression, the expression of pRb in cells transfected with miR-154-5p mimic was higher than mimic NC, while the expression of pRb in cells transfected with the miR-154-5p inhibitor was lower than inhibitor NC (both *P<*0.05, Figure 5C, 5D). In addition, Pearson correlation analysis showed an inverse correlation between the CUL2 and pRb expression (*r* = -0.879, *P* < 0.001, Figure 5E).

### Knock-down of CUL2 suppressed proliferation, migration, and invasion of SiHa cells and increased pRb expression

**Figure 6:**
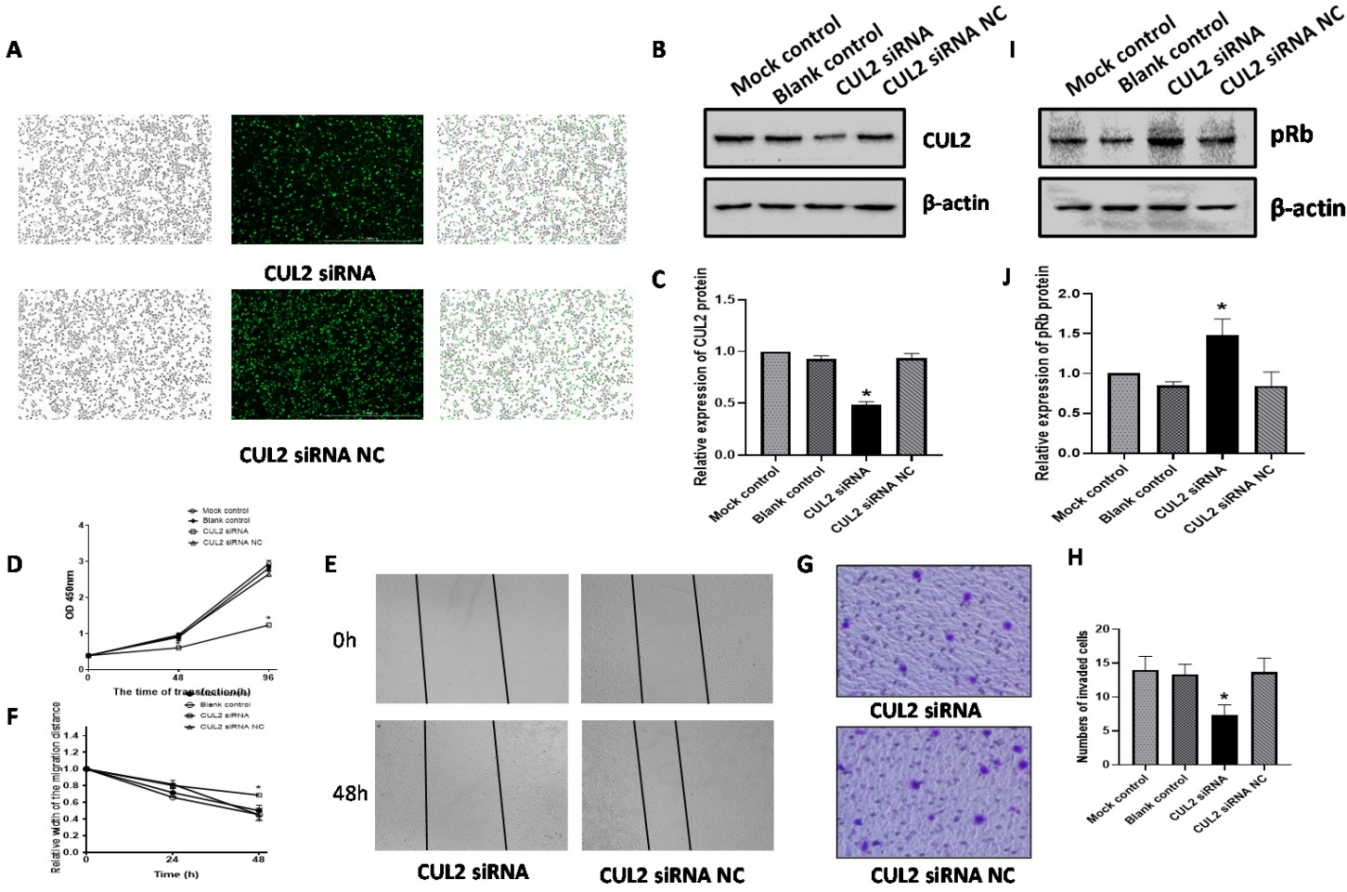
** CUL2 effected SiHa cells proliferation, migration, invasion, and the expression of pRb.** (A) SiHa cells were transfected with CUL2 siRNA or siRNA NC; (B-C) The relative expression of CUL2 protein 72h post transfection; (D) CUL2 effected the SiHa cells proliferation; (E-F) CUL2 effected the SiHa cells migration; (G-H) CUL2 effected the SiHa cells invasion; (I-J) CUL2 regulated the expression of pRb and the relative expression of protein was measured by gray value assays. (*, P<0.05 versus corresponding NC).

We transfected CUL2 siRNA into SiHa cells and assessed the effect on proliferation, migration, and invasion. Fluorescence microscopy detection showed that transfection efficiencies were over 90% (Figure 6A). Western blot analysis showed that at 72h following transfection, CUL2 protein levels were significantly decreased in cells transfected with CUL2 siRNA compared with siRNA NC (*P<*0.001, Figure 6B, 6C).

CUL2 protein knockdown reduced SiHa cells proliferation at both 48h and 96h following transfection with CUL2 siRNA (both *P<*0.05, Figure 6D). Further, CUL2 siRNA considerably inhibited SiHa cells migration when compared to the siRNA NC at 48 h post transfection (*P<*0.05, Figure 6E, 6F). The invasive ability of SiHa cells in the CUL2 siRNA group decreased by 46.34% compared with siRNA NC (*P*=0.0112, Figure 6G, 6H). At 72 h after transfection of SiHa cells with CUL2 siRNA, the pRb expression was higher than siRNA NC (*P<*0.05, Figure 6I, 6J).

## Discussion

Cervical cancer accounts for 570,000 new cases and 311,000 deaths worldwide annually, more than those of any other gynecological tumor 9. SCC is the most prevalent cervical cancer and is closely associated with the carcinogenic HPV16 type. Nearly half the women with HPV16 infections persisting for at least two years developed precancer lesions within the subsequent five years 10. The *E6* and *E7* genes are two known key oncogenes of HPV16. Compared to *E6*, more HPV16 *E7* is required to sustain a malignant phenotype during an extensive characterization of primary cervical cancer HPV transcripts 11, making it a more potent driver for cervical cancer 12. The E7 oncoprotein binds preferentially to the tumor suppressor pRb and disrupts the pRb/E2F transcriptional repressor complex to initiate oncogenic transformation, leading to dysregulation of epithelial cell growth, which eventually leads to CIN progression, ultimately causing cervical cancer 13, 14. Therefore, blocking HPV16 E7-mediated pRb degradation may inhibit the progression of CIN to SCC caused by persistent HPV16 infection.

The ubiquitin proteasome system is an important pathway for protein degradation in cellular organisms, which composed of ubiquitin, E1 ubiquitin-activating enzyme, E2 ubiquitin-binding enzyme, E3 ubiquitin ligase, and the proteasome. Cullin-Ring E3 ubiquitin ligases are the largest family of E3 ubiquitin ligases and are widely involved in the regulation of cyclins and transcription factor degradation 15. Among these, CUL2 ubiquitin ligase complex has been shown to regulate cell cycle and tumorigenesis and become a hot topic of research 16, 17. As the core component of the E3 ubiquitin-protein ligase complex, CUL2 has been predicted to be a tumor-suppressor since it causes the degradation of the α subunits of the pro-oncogenic hypoxia inducible factor 17. In contrast, CUL2 has been reported to promote the progression of gastric cancer 18. In the present study, we showed that inhibition of CUL2 expression significantly suppressed the oncogenic properties of SiHa cells. These results support an oncogenic role for CUL2 in the development of HPV16-induced cervical cancer, consistent with findings from a previous study 19. In addition, we found a strong inverse correlation between the CUL2 and pRb levels *in vitro*. It has been reported that depletion of CUL2 by RNA interference increased the steady-state levels and stability of pRb in HPV16 E7-expressing cells rather than increasing translation of pRb 5. Therefore, CUL2-mediated regulation of HPV16 E7-induced pRb degradation is a critical step for the progression of HPV16 infection into cervical cancer. However, the regulation of CUL2 is poorly understood.

The association between miRNA expression and cervical tumorigenesis has been investigated in epigenetic regulation studies. A systematic study of miRNA profiles from CIN to cervical cancer identified 42 up regulated and 21 down regulated miRNAs among different stages of cervical cancer development 20. Approximately 50% of miRNA genes are located in fragile sites that are preferential targets for HPV16 integrations in cervical tumors 21. Based on this, miRNAs play a synergic role in HPV16 infection leading to cervical cancer. Recent studies have found that in cervical cancer cells, miR-424 binds to CUL2 mRNA, and that overexpression of miR-424 decreases CUL2 mRNA and protein expression, thereby inhibiting cell proliferation, migration and invasion, promoting cell cycle arrest in the G1/S phase 19. This suggests that the miRNA-mediated CUL2 regulation is an important target for blocking cervical cancer. However, reports on the correlation between miRNA expression and CUL2 regulation in the context of cervical cancer are rare. Therefore, insight into the regulatory networks of miRNA that target CUL2 during CIN progression may help to identify their contribution to the pathogenesis of cervical cancer.

In this study, we have identified a total of 77 miRNAs differentially expressed in CIN3 and SCC tissues compared with normal cervix tissues. According to GO analysis, “protein ubiquitination involved in ubiquitin-dependent protein catabolic processes” (GO: 0042787) had the second highest enrichment score, suggesting that this pathway is important for regulating protein levels and function during the development of HPV16-induced cervical cancer. The CUL2 scaffold protein is required for ubiquitin-dependent protein degradation 22. In our study, bioinformatics analysis predicted that the CUL2 3'UTR was potentially targeted by the 17 differentially expressed miRNAs, among which miR-154-5p had the highest binding context score. Furthermore, the dual-luciferase reporter assay confirmed that miR-154-5p directly targeted CUL2 through its 3'UTR. Our findings indicate that CUL2 is a downstream target of miR-154-5p.

miR-154-5p is located on human chromosome 14q32 and is involved in the pathophysiology of various disorders 23. Recently it has been shown that miR-154-5p, which is involved as a suppressor in the regulation of cancers, was downregulated in breast cancer, colorectal cancer, glioma, hepatocellular cancer, osteosarcoma, nasopharyngeal carcinoma, and non-small cell lung cancer 24-31. Intriguingly, Lin *et al*. revealed that miR-154-5p was up regulated in renal cell carcinoma and acted as an oncogene with poor prognosis 32. These results suggest that miR-154-5p may play a tumor-suppressive or oncogenic role, depending on the tumor types. Nevertheless, there are no studies on the expression of miR-154-5p and its role in cervical cancer. To our knowledge, this is the first report which shows that miR-154-5p expression was significantly reduced in CIN and SCC tissues. Therefore, we further investigated the miR-154-5p functions in the progression of cervical cancer. Our data showed that enforced expression of miR-154-5p significantly reduced the proliferation, migration, and invasion ability of SiHa cells. Conversely, inhibition of miR-154-5p had the opposite effect. Thus, the downregulation of miR-154-5p may be one of the factors that acts in coordination with HPV16 infection to induce cervical carcinogenesis or cervical cancer progression.

miRNA functions are observed through their effects on specific target genes. A recent study showed that miR-154-5p inhibits glioma cell proliferation, migration, and invasion by regulating PIWIL1 genes 29. In colorectal cancer, miR-154-5p inhibits tumor cell proliferation and migration by blocking CCND2 33. However, the target gene(s) of miR-154-5p in cervical cancer have not yet been identified. As mentioned previously, we used bioinformatics analysis to predict and the dual-luciferase reporter assay to confirm that CUL2 is a target of miR-154-5p in cervical cancer. Further, the western blot assay showed that miR-154-5p overexpression inhibited the CUL2 protein expression, while miR-154-5p knockdown promoted the CUL2 protein expression. Meanwhile, our data showed a critical role for miR-154-5p targeting CUL2 during cervical carcinogenesis, which is mediated through pRb expression. We showed that overexpression of miR-154-5p inhibited the oncogenic properties of SiHa cells via decreasing CUL2 and increasing pRb expression. Conversely, miR-154-5p knockdown promoted the oncogenic properties of SiHa cells via increasing CUL2 and decreasing pRb expression.

In conclusion, our study indicated that miR-154-5p plays a crucial role in the inhibition of HPV16 E7-mediated pRb degradation by targeting the CUL2 3'UTR. Our results suggest that miR-154-5p is a potential target for the treatment of HPV-induced cervical cancer.

## Supplementary Material

Supplementary table.

## Figures and Tables

**Table 1:** Baseline characteristics of the subjects for microarray analysis

Group	Sample NO.	Pathology	Age	HPV type	TCT
1	0000134	Normal cervix	46	16	NILM
1	2100365	Normal cervix	44	16	NILM
1	2100071	Normal cervix	47	16	NILM
1	2100371	Normal cervix	43	16	NILM
1	2100222	Normal cervix	45	16	NILM
2	0000219	CIN3	45	16	ASCUS
2	2100218	CIN3	50	16	HSIL
2	2100209	CIN3	46	16	ASC-H
2	0000043	CIN3	42	16	ASCUS
2	2100399	CIN3 with gland involvement	50	16	ASC-H
2	2100046	CIN3 with gland involvement	48	16	NILM
2	0000126	CIN3	43	16	LSIL
2	2100005	CIN3	46	16	ASCUS
2	0000179	CIN3	41	16	ASCUS
2	2100068	CIN3	43	16	NILM
3	2100279	SCC G2	48	16	SCC
3	2100387	SCC G2	49	16	HSIL
3	2100418	SCC G2	46	16	ASCUS
3	0000266	SCC G2	50	16	ASCUS
3	0000172	SCC G2	44	16	SCC
3	2100125	SCC G2	46	16	ASCUS
3	2100385	SCC G2	50	16	HSIL
3	0000048	SCC G2	47	16	NILM
3	0000104	SCC G2	50	16	ASCUS
3	2100290	SCC G2	49	16	SCC

TCT, ThinPrep cytologic test; NILM, negative for intraepithelial lesion or malignancy; ASCUS, atypical squamous cells of undetermined significance; ASC-H, atypical cells of undetermined significance, cannot exclude a high-grade squamous intraepithelial lesion; LSIL, low-grade squamous intraepithelial lesion; HSIL, high-grade squamous intraepithelial lesion; SCC, squamous cell carcinoma.

**Table 2:** The sequences of primers and oligonucleotides used in this study

Primer/Oligonucleotide	Sequence (5'-3')
miR-154-5p F	GCGCGCGTAGGTTATCCGTGTTG
miR-154-5p R	ATCCAGTGCAGGGTCCGAGG
miR-154-5p RT	GTCGTATCCAGTGCAGGGTCCGAGGTATTCGCACTGGATACGACCGAAGG
H-U6 F	CTCGCTTCGGCAGCACA
H-U6 R	AACGCTTCACGAATTTGCGT
H-U6 RT	AACGCTTCACGAATTTGCGT
miR-154-5p mimic sense	UAGGUUAUCCGUGUUGCCUUCG
miR-154-5p mimic anti-sense	AAGGCAACACGGAUAACCUAUU
miR-154-5p mimic NC sense	UUCUCCGAACGUGUCACGUTT
miR-154-5p mimic NC anti-sense	ACGUGACACGUUCGGAGAATT
miR-154-5p inhibitor sense	CGAAGGCAACACGGAUAACCUA
miR-154-5p inhibitor NC sense	CAGUACUUUUGUGUAGUACAA
CUL2 siRNA sense	CCUCUUACUCAGGCUCCUUTT
CUL2 siRNA anti-sense	AAGGAGCCUGAGUAAGAGGTT
CUL2 siRNA NC sense	UUCUCCGAACGUGUCACGUTT
CUL2 siRNA NC anti-sense	ACGUGACACGUUCGGAGAATT

**Table 3:** 3'-UTR of CUL2 was potentially targeted by 17 differentially expressed miRNAs in this study

miRNA	Position in the UTR	seed match	context++ score	context++ score percentile	weighted context++ score	conserved branch length
hsa-miR-93-3p	705-711	7mer-m8	-0.14	86	-0.04	0.072
hsa-miR-199a-3p	1605-1611	7mer-1A	-0.16	84	-0.05	1.151
hsa-miR-183-3p	551-557	7mer-m8	-0.02	59	-0.01	0.072
hsa-miR-199b-3p	1605-1611	7mer-1A	-0.16	84	-0.05	1.151
hsa-miR-224-5p	1620-1626	7mer-1A	-0.12	88	-0.03	1.926
hsa-miR-27b-5p	181-187	7mer-1A	-0.2	89	-0.2	1.671
hsa-miR-154-5p	373-380	8mer	-0.44	99	-0.44	5.685
	1008-1015	8mer	-0.21	93	-0.06	0.851
hsa-miR-195-5p	417-423	7mer-m8	-0.34	97	-0.27	8.638
hsa-miR-424-5p	417-423	7mer-m8	-0.41	98	-0.33	8.638
hsa-miR-494-3p	1268-1274	7mer-m8	-0.02	51	-0.01	0.192
hsa-miR-654-3p	734-740	7mer-m8	-0.03	49	-0.01	0.075
hsa-miR-1290	1447-1454	8mer	-0.22	94	-0.06	0.021
hsa-miR-1246	827-833	7mer-m8	-0.14	86	-0.04	0.021
hsa-miR-1914-3p	1204-1210	7mer-m8	-0.11	83	-0.03	0
hsa-miR-4443	81-87	7mer-m8	-0.28	94	-0.28	0.901
	949-955	7mer-1A	-0.13	80	-0.04	0
hsa-miR-4738-3p	709-715	7mer-m8	-0.02	44	-0.01	0
hsa-miR-6825-5p	920-926	7mer-m8	-0.23	78	-0.06	0

## Abbreviations

CIN: cervical intraepithelial neoplasia
GO: Gene ontology
KEGG: Kyoto Encyclopedia of Genes and Genomes
TCT: ThinPrep cytologic test
NILM: negative for intraepithelial lesion or malignancy
ASCUS: atypical squamous cells of undetermined significance
ASC-H: atypical cells of undetermined significance, cannot exclude a high-grade squamous intraepithelial lesion
LSIL: low-grade squamous intraepithelial lesion
HSIL: high-grade squamous intraepithelial lesion
SCC: squamous cell carcinoma
CUL2: Cullin 2
pRb: retinoblastoma protein
FIGO: International Federation of Gynecology and Obstetrics
ANOVA: analysis of variance

## References

[1] Siegel RL, Miller KD, Jemal A (2019). Cancer statistics, 2019. CA Cancer J Clin.

[2] McClung NM, Gargano JW, Bennett NM (2019). Trends in Human Papillomavirus Vaccine Types 16 and 18 in Cervical Precancers, 2008-2014. Cancer Epidemiol Biomarkers Prev.

[3] Zhang C, Luo Y, Zhong R (2019). Role of polycyclic aromatic hydrocarbons as a co-factor in human papillomavirus-mediated carcinogenesis. BMC Cancer.

[4] Zhao W, Hao M, Wang Y (2016). Association between folate status and cervical intraepithelial neoplasia. Eur J Clin Nutr.

[5] Huh K, Zhou X, Hayakawa H (2007). Human papillomavirus type 16 E7 oncoprotein associates with the cullin 2 ubiquitin ligase complex, which contributes to degradation of the retinoblastoma tumor suppressor. J Virol.

[6] White EA, Sowa ME, Tan MJ (2012). Systematic identification of interactions between host cell proteins and E7 oncoproteins from diverse human papillomaviruses. Proc Natl Acad Sci U S A.

[7] Todorovic B, Hung K, Massimi P (2012). Conserved region 3 of human papillomavirus 16 E7 contributes to deregulation of the retinoblastoma tumor suppressor. J Virol.

[8] Miroshnichenko S, Patutina O (2019). Enhanced Inhibition of Tumorigenesis Using Combinations of miRNA-Targeted Therapeutics. Front Pharmacol.

[9] Arbyn M, Weiderpass E, Bruni L (2020). Estimates of incidence and mortality of cervical cancer in 2018: a worldwide analysis. Lancet Glob Health.

[10] Kjaer SK, Frederiksen K, Munk C (2010). Long-term absolute risk of cervical intraepithelial neoplasia grade 3 or worse following human papillomavirus infection: role of persistence. J Natl Cancer Inst.

[11] Cancer Genome Atlas Research N, Albert Einstein College of M, Analytical Biological S (2017). Integrated genomic and molecular characterization of cervical cancer. Nature.

[12] Mirabello L, Yeager M, Yu K (2017). HPV16 E7 Genetic Conservation Is Critical to Carcinogenesis. Cell.

[13] Songock WK, Kim SM, Bodily JM (2017). The human papillomavirus E7 oncoprotein as a regulator of transcription. Virus Res.

[14] Hoppe-Seyler K, Bossler F, Braun JA (2018). The HPV E6/E7 Oncogenes: Key Factors for Viral Carcinogenesis and Therapeutic Targets. Trends Microbiol.

[15] Scott DC, Rhee DY, Duda DM (2016). Two Distinct Types of E3 Ligases Work in Unison to Regulate Substrate Ubiquitylation. Cell.

[16] Wang S, Xia W, Qiu M (2016). Atlas on substrate recognition subunits of CRL2 E3 ligases. Oncotarget.

[17] Cai W, Yang H (2016). The structure and regulation of Cullin 2 based E3 ubiquitin ligases and their biological functions. Cell Div.

[18] Su Y, Ni Z, Wang G (2012). Aberrant expression of microRNAs in gastric cancer and biological significance of miR-574-3p. Int Immunopharmacol.

[19] Xu J, Fang Y, Wang X (2016). CUL2 overexpression driven by CUL2/E2F1/miR-424 regulatory loop promotes HPV16 E7 induced cervical carcinogenesis. Oncotarget.

[20] He Y, Lin J, Ding Y (2016). A systematic study on dysregulated microRNAs in cervical cancer development. Int J Cancer.

[21] Gomez-Gomez Y, Organista-Nava J, Gariglio P (2013). Deregulation of the miRNAs expression in cervical cancer: human papillomavirus implications. Biomed Res Int.

[22] Cardote TAF, Gadd MS, Ciulli A (2017). Crystal Structure of the Cul2-Rbx1-EloBC-VHL Ubiquitin Ligase Complex. Structure.

[23] Ren H, Ma X, Shao Y (2019). Correlation Between Serum miR-154-5p and Osteocalcin in Males and Postmenopausal Females of Type 2 Diabetes With Different Urinary Albumin Creatinine Ratios. Front Endocrinol (Lausanne).

[24] Bolandghamat Pour Z, Nourbakhsh M, Mousavizadeh K (2019). Suppression of nicotinamide phosphoribosyltransferase expression by miR-154 reduces the viability of breast cancer cells and increases their susceptibility to doxorubicin. BMC Cancer.

[25] Kai Y, Qiang C, Xinxin P (2015). Decreased miR-154 expression and its clinical significance in human colorectal cancer. World J Surg Oncol.

[26] Wang L, Wu L, Wu J (2016). Downregulation of miR-154 in human glioma and its clinicopathological and prognostic significance. J Int Med Res.

[27] Lin X, Yang Z, Zhang P (2015). miR-154 suppresses non-small cell lung cancer growth *in vitro* and *in vivo*. Oncol Rep.

[28] Pang X, Huang K, Zhang Q (2015). miR-154 targeting ZEB2 in hepatocellular carcinoma functions as a potential tumor suppressor. Oncol Rep.

[29] Zhou H, Zhang Y, Lai Y (2020). Circ_101064 regulates the proliferation, invasion and migration of glioma cells through miR-154-5p/ PIWIL1 axis. Biochem Biophys Res Commun.

[30] Chen J, Ma C, Zhang Y (2020). MiR-154-5p Suppresses Cell Invasion and Migration Through Inhibiting KIF14 in Nasopharyngeal Carcinoma. Onco Targets Ther.

[31] Tian Q, Gu Y, Wang F (2020). Upregulation of miRNA-154-5p prevents the tumorigenesis of osteosarcoma. Biomed Pharmacother.

[32] Lin C, Li Z, Chen P (2018). Oncogene miR-154-5p regulates cellular function and acts as a molecular marker with poor prognosis in renal cell carcinoma. Life Sci.

[33] Xu M, Chen X, Lin K (2018). The long noncoding RNA SNHG1 regulates colorectal cancer cell growth through interactions with EZH2 and miR-154-5p. Mol Cancer.

