# Supplementary material for: MicroRNA-154-5p regulates the HPV16 E7-pRb pathway in Cervical Carcinogenesis by targeting CUL2
Source: J Cancer. 2020 Jul 11;11(18):5379–89. doi: 10.7150/jca.45871 (PMC7391205; doi:10.7150/jca.45871)
Supplement: Supplementary file 1 — Supplementary table. [file jcav11p5379s1.pdf]

Supplementary Table 1. Differently expressed miRNAs in normal cervix, CIN3 and SCC tissues.

Transcript Cluster ID	Normal cervix	CIN3	SCC	FC	ANOVA p-value	Transcript ID	Accession	Sequence Length	Sequence
20500132	5.84	5.7	6.97	2.411615655	0.011473	hsa-miR-18a-5p	MIMAT0000072	23	UAAGGUGCAUCUAGUGCAGAUAG
20500133	2.54	2.63	4.11	2.969047141	0.009958	hsa-miR-18a-3p	MIMAT0002891	23	ACUGCCCUAAGUGCUCCUUCUGG
20500142	3.63	4.51	5.47	3.580100284	0.002311	hsa-miR-21-3p	MIMAT0004494	21	CAACACCAGUCGAUGGGCUGU
20500145	4.56	4.65	5.75	2.281527432	0.010072	hsa-miR-23a-5p	MIMAT0004496	22	GGGGUUCCUGGGGAUGGGAUUU
20500150	3.72	4.42	5.32	3.031433133	0.000513	hsa-miR-25-5p	MIMAT0004498	21	AGGCGGAGACUUGGGCAAUUG
20500156	2.51	3.86	5.64	8.75434961	0.000411	hsa-miR-27a-5p	MIMAT0004501	22	AGGGCUUAGCUGCUUGUGAGCA
20500170	1.27	2.6	4.22	7.727490631	0.000473	hsa-miR-92a-1-5p	MIMAT0004507	23	AGGUUGGGAUCGGUUGCAAUGCU
20500174	4.83	4.79	5.87	2.114036081	0.023707	hsa-miR-93-3p	MIMAT0004509	22	ACUGCUGAGCUAGCACUUCCCG
20500181	11.73	11.29	9.42	4.9588308	0.006134	hsa-miR-99a-5p	MIMAT0000097	22	AACCCGUAGAUCCGAUCUUGUG
20500183	10.45	10.45	8.66	3.458148925	0.047895	hsa-miR-100-5p	MIMAT0000098	22	AACCCGUAGAUCCGAACUUGUG
20500393	1.54	1.28	5.53	19.02731384	0.00429	hsa-miR-196a-5p	MIMAT0000226	22	UAGGUAGUUUCAUGUUGUUGGG
20500400	10.32	10.76	9.28	2.789487333	0.012829	hsa-miR-199a-3p	MIMAT0000232	22	ACAGUAGUCUGCACAUUGGUUA
20500438	7.28	8.24	6.04	4.59479342	0.019024	hsa-miR-10a-5p	MIMAT0000253	23	UACCCUGUAGAUCCGAAUUUGUG
20500450	8.22	8.53	9.25	2.042024251	0.038977	hsa-miR-182-5p	MIMAT0000259	24	UUUGGCAAUGGUAGAACUCACACU
20500452	4.59	5.97	6.62	4.084048503	0.009666	hsa-miR-183-5p	MIMAT0000261	22	UAUGGCACUGGUAGAAUUCACU
20500453	1.11	1.71	3.26	4.438277888	0.010949	hsa-miR-183-3p	MIMAT0004560	22	GUGAAUUACCGAAGGGCCAUAA
20500457	4.69	4.65	1.86	7.110741449	0.034627	hsa-miR-199b-5p	MIMAT0000263	23	CCCAGUGUUUAGACUAUCUGUUC
20500458	10.32	10.76	9.28	2.789487333	0.012829	hsa-miR-199b-3p	MIMAT0004563	22	ACAGUAGUCUGCACAUUGGUUA
20500463	0.91	0.85	2.67	3.530811985	0.031006	hsa-miR-205-3p	MIMAT0009197	21	GAUUUCAGUGGAGUGAAGUUC
20500489	4.6	5.63	6.55	3.863745316	0.029366	hsa-miR-224-5p	MIMAT0000281	21	CAAGUCACUAGUGGUUCCGUU
20500555	6.25	7.91	7.46	3.160165247	0.029931	hsa-miR-200b-5p	MIMAT0004571	22	CAUCUUACUGGGCAGCAUUGGA
20500556	7.5	9.13	8.4	3.095129987	0.046326	hsa-miR-200b-3p	MIMAT0000318	22	UAAUACUGCCUGGUAAUGAUGA
20500722	4.01	5.01	5.77	3.386981249	0.003796	hsa-miR-27b-5p	MIMAT0004588	22	AGAGCUUAGCUGAUUGGUGAAC
20500730	12.58	12.57	11.15	2.694467154	0.003039	hsa-miR-125b-5p	MIMAT0000423	22	UCCCUGAGACCCUAACUUGUGA
20500784	3.37	3.18	0.76	6.105036836	0.002713	hsa-miR-154-5p	MIMAT0000452	22	UAGGUUAUCCGUGUUGCCUUCG
20500798	10.68	10.9	8.87	4.084048503	0.000612	hsa-miR-195-5p	MIMAT0000461	21	UAGCAGCACAGAAAUAUUGGC
20501159	3.14	2.49	1.55	3.010493495	0.048142	hsa-miR-29c-5p	MIMAT0004673	22	UGACCGAUUUCUCCUGGUGUUC
20501172	1.08	1.98	0.64	2.531513188	0.024299	hsa-miR-299-5p	MIMAT0002890	22	UGGUUUACCGUCCCACAUACAU
20501224	4.76	4.67	2.05	6.543216468	0.0358	hsa-miR-376c-3p	MIMAT0000720	21	AACAUAGAGGAAAUUCCACGU

20501249	2.94	2.76	1.08	3.630076621	0.038799	hsa-miR-381-3p	MIMAT0000736	22	UAUACAAGGGCAAGCUCUCUGU
20501281	4.72	3.77	2.02	6.498019171	0.032777	hsa-miR-337-5p	MIMAT0004695	21	GAACGGCUUCAUACAGGAGUU
20502125	2.8	3.54	1.21	5.028053498	0.006824	hsa-miR-424-5p	MIMAT0001341	22	CAGCAGCAAUUCAUGUUUUGAA
20502235	1.79	2.34	3.9	4.316912946	0.001094	hsa-miR-18b-5p	MIMAT0001412	23	UAAGGUGCAUCUAGUGCAGUUAG
20502441	2.6	3.96	1.19	6.821079134	0.010122	hsa-miR-433-3p	MIMAT0001627	22	AUCAUGAUGGGCUCCUCGGUGU
20503803	8.1	7.73	7.09	2.0139111	0.021848	hsa-miR-494-3p	MIMAT0002816	22	UGAAACAUACACGGGAAACCUC
20503877	1.93	2.83	3.25	2.496661098	0.025726	hsa-miR-501-5p	MIMAT0002872	22	AAUCCUUUGUCCCUGGGUGAGA
20504325	1.8	2.85	3.45	3.138336392	0.039014	hsa-miR-550a-3p	MIMAT0003257	22	UGUCUUACUCCCUCAGGCACAU
20504353	0.82	1.61	2.03	2.313376368	0.038855	hsa-miR-612	MIMAT0003280	25	GCUGGGCAGGGCUUCUGAGCUCCUU
20504357	0.33	0.41	1.82	2.808889751	0.006142	hsa-miR-615-3p	MIMAT0003283	22	UCCGAGCCUGGGUCUCCCUCUU
20504362	5.89	3.7	1.12	27.28431654	0.040973	hsa-miR-617	MIMAT0003286	22	AGACUUCCCAUUUGAAGGUGGC
20504390	1.49	2.72	2.37	2.345669898	0.013819	hsa-miR-637	MIMAT0003307	24	ACUGGGGGCUUUCGGGCUCUGCGU
20504407	1.14	2.22	2.38	2.361985323	0.004941	hsa-miR-652-5p	MIMAT0022709	25	CAACCCUAGGAGAGGGUGCCAUUCA
20504421	4.11	3.9	1.48	6.190259974	0.007056	hsa-miR-654-3p	MIMAT0004814	22	UAUGUCUGCUGACCAUCACCUU
20504434	2.03	3.01	0.72	4.890561111	0.045974	hsa-miR-542-5p	MIMAT0003340	23	UCGGGGAUCAUCAUGUCACGAGA
20504556	2.06	3.06	3.26	2.29739671	0.009567	hsa-miR-550a-3-5p	MIMAT0020925	20	AGUGCCUGAGGGAGUAAGAG
20505789	0.96	2.6	0.75	3.60500185	0.047856	hsa-miR-885-5p	MIMAT0004947	22	UCCAUUACACUACCCUGCCUCU
20506002	3.41	4.67	4.58	2.394957409	0.016407	hsa-miR-933	MIMAT0004976	22	UGUGCGCAGGGAGACCUCUCCC
20506818	1.18	1.73	4.73	11.71268557	0.001508	hsa-miR-1290	MIMAT0005880	19	UGGAUUUUUGGAUCAGGGA
20506837	7.1	7.75	8.8	3.249009585	0.009619	hsa-miR-1246	MIMAT0005898	19	AAUGGAUUUUUGGAGCAGG
20506887	1.92	2.64	3.04	2.173469725	0.000435	hsa-miR-1292-5p	MIMAT0005943	25	UGGGAACGGGUUCCGGCAGACGCUG
20509232	1.77	2.06	0.44	3.073750363	0.027465	hsa-miR-1912	MIMAT0007887	22	UACCCAGAGCAUGCAGUGUGAA
20509235	0.97	2.3	2.01	2.514026749	0.010508	hsa-miR-1914-3p	MIMAT0007890	22	GGAGGGGUCCCGCACUGGGAGG
20515607	8.5	9.48	9.68	2.265767771	0.002746	hsa-miR-3178	MIMAT0015055	17	GGGGCGCGGCCGGAUCG
20515610	5.38	6.39	6.43	2.070529848	0.010024	hsa-miR-3180-3p	MIMAT0015058	22	UGGGGCGGAGCUUCCGGAGGCC
20515623	3.09	4.01	4.11	2.02791896	0.013168	hsa-miR-3187-3p	MIMAT0015069	20	UUGGCCAUGGGGCUGCGCGG
20517817	0.49	1.87	1.52	2.602683711	0.005233	hsa-miR-3610	MIMAT0017987	20	GAAUCGGAAAGGAGGCGCCG
20517821	6.53	7.32	7.99	2.751083636	0.02787	hsa-miR-3613-3p	MIMAT0017991	24	ACAAAAAAAAAAGCCCAACCCUUC
20517824	2.25	3.33	3.48	2.345669898	0.005343	hsa-miR-3615	MIMAT0017994	21	UCUCUCGGCUCCUCGCGGCUC
20517916	5	5.67	6.23	2.345669898	0.008639	hsa-miR-3663-3p	MIMAT0018085	23	UGAGCACCACACAGGCCGGGCGC
20518785	4.9	6.64	6.45	3.340351678	0.038644	hsa-miR-4417	MIMAT0018929	18	GGUGGGCUUCCCGGAGGG
20518818	6.23	6.88	7.36	2.188587403	0.029035	hsa-miR-4443	MIMAT0018961	17	UUGGAGGCGUGGGUUUU

20518844	5.67	5.57	4.42	2.37841423	0.046067	hsa-miR-4462	MIMAT0018986	23	UGACACGGAGGGUGGCUUGGGAA
20518919	4.91	4.76	6.43	3.182145935	0.006492	hsa-miR-4521	MIMAT0019058	22	GCUAAGGAAGUCCUGUGCUCAG
20519573	3.83	4.97	4.7	2.203810232	0.030294	hsa-miR-4730	MIMAT0019852	23	CUGGCGGAGCCCAUUCCAUGCCA
20519588	2.33	3.82	3.77	2.808889751	0.025629	hsa-miR-4738-3p	MIMAT0019867	22	UGAAACUGGAGCGCCUGGAGGA
20519670	1.75	3.22	3.29	2.907945035	0.000201	hsa-miR-4783-3p	MIMAT0019947	23	CCCCGGUGUUGGGGCGCGUCUGC
20525414	3.44	4.6	4.86	2.67585511	0.024649	hsa-miR-6726-5p	MIMAT0027353	21	CGGGAGCUGGGGUCUGCAGGU
20525432	2.51	3.88	3.39	2.584705661	0.016537	hsa-miR-6735-5p	MIMAT0027371	25	CAGGGCAGAGGGCACAGGAAUCUGA
20525442	1.59	2.64	2.46	2.070529848	0.046065	hsa-miR-6740-5p	MIMAT0027381	22	AGUUUGGGAUGGAGAGAGGAGA
20525509	3.47	4.56	4.55	2.128740365	0.001692	hsa-miR-6774-5p	MIMAT0027448	25	ACUUGGGCAGGAGGGACCCUGUAUG
20525611	2.01	3.57	2.92	2.948538435	0.039101	hsa-miR-6825-5p	MIMAT0027550	22	UGGGGAGGUGUGGAGUCAGCAU
20525633	4.46	5.12	5.77	2.4794154	0.005709	hsa-miR-6780b-5p	MIMAT0027572	23	UGGGGAAGGCUUGGCAGGGAAGA
20525653	3.21	4.59	4.44	2.602683711	0.010016	hsa-miR-6846-5p	MIMAT0027592	22	UGGGGGCUGGAUGGGGUAGAGU
20525701	3.9	4.71	5.08	2.265767771	0.017735	hsa-miR-6870-5p	MIMAT0027640	19	UGGGGGAGAUGGGGGUUGA
20525731	1.13	2.26	2.16	2.188587403	0.044783	hsa-miR-6885-5p	MIMAT0027670	25	AGGGGGGCACUGCGCAAGCAAAGCC
20526180	3.94	5.03	4.71	2.128740365	0.024455	hsa-miR-7111-5p	MIMAT0028119	22	UGGGGGAGGAAGGACAGGCCAU
20526182	2.29	3.45	3.64	2.549121255	0.014317	hsa-miR-7112-5p	MIMAT0028121	22	ACGGGCAGGGCAGUGCACCCUG

Fold Change cut-off: 2.0
# P-value cut-off: 0.05
Conditions: cervical cancer (SCC)_vs_CIN3_vs_Normal cervix
Column A: Transcript Cluster ID, it represents transcript cluster name.
Column B - D: Bi-weight Avg Signal (log2), Normalized Intensity of each group (averaged normalized intensities of each group, log2 transformed)
Column E: FC, the absolute ratio (no log scale) of normalized intensities between three conditions.
Column F: ANOVA P value (All Conditions),   P value calculated from the student’s t-test.
Column G: Transcript ID (Array Design).
Column H: Accession.
Column I: Sequence Length.
Column J: Sequence from miRBase.
